# Fatty liver index in young adult offspring of women with type 1 diabetes

**DOI:** 10.1186/s13098-023-01164-0

**Published:** 2023-10-11

**Authors:** Cedric A. Korpijaakko, Johan G. Eriksson, Hannu Kautiainen, Miira M. Klemetti, Merja K. Laine

**Affiliations:** 1 Department of General Practice and Primary Health Care, University of Helsinki and Helsinki University Hospital, Helsinki, Finland; 2 Folkhälsan Research Center, Helsinki, Finland; 3 Human Potential Translational Research Programme and Department of Obstetrics and Gynecology, Yong Loo Lin School of Medicine, National University Singapore, Singapore, Singapore; 4 Singapore Institute for Clinical Sciences (SICS), Agency for Science, Technology and Research (A*STAR), Singapore, Singapore; 5 Primary Health Care Unit, Kuopio University Hospital, Kuopio, Finland; 6 Department of Obstetrics and Gynecology, Helsinki University Hospital and University of Helsinki, Helsinki, Finland; 7 Department of Medical and Clinical Genetics, University of Helsinki, Helsinki, Finland

**Keywords:** Fatty liver index, Offspring, Nonalcoholic fatty liver disease, Type 1 diabetes

## Abstract

**Background:**

Exposure to a hyperglycemic environment during prenatal life may result in an unfavorable metabolic profile later in adulthood. We aimed to assess whether fatty liver index, a non-invasive indicator of nonalcoholic fatty liver disease risk, differs in young adult offspring of women with type 1 diabetes from offspring of women without diabetes.

**Methods:**

This cohort study was conducted within the hospital district of Helsinki and Uusimaa, Finland. Between 1996 and 2000, we identified 238 singleton offspring of women with type 1 diabetes, born at the Department of Obstetrics and Gynecology, Helsinki University Hospital, Helsinki, Finland. From the Finnish Medical Birth Register, we identified 476 singleton age- and region-matched offspring of women without diabetes. At 18–23 years of age, 70 offspring of women with type 1 diabetes and 83 offspring of women without diabetes participated in a clinical study, including laboratory tests, clinical assessments, and self-reported questionnaires. The noninvasive fatty liver index was used to estimate nonalcoholic fatty liver disease.

**Results:**

Fatty liver index (FLI) was similar between offspring of women with type 1 diabetes and offspring of women without diabetes (p = 0.59). Additionally, no differences between the groups could be observed for FLI ≥ 60, i.e., to cut-off value for NAFLD. Likewise, we could not find any statistically significant differences between young adult offspring of women with type 1 diabetes (20.4 years [SD 1.6]) and young adult offspring of women without diabetes (20.6 years [SD 1.6]) regarding metabolic characteristics: BMI 24.5 kg/m^2^ vs. 24.0 kg/m^2^, fasting plasma glucose 5.39 mmol/L vs. 5.40 mmol/L, fasting insulin 11.0 mU/L vs. 10.6 mU/L, total cholesterol 4.36 mmol/L vs. 4.30 mmol/L, systolic BP 117 mmHg vs. 119 mmHg, triglycerides 0.89 mmol/L vs. 0.96 mmol/L, and Waist-to-height ratio 0.41 vs. 0.42.

**Conclusions:**

Our results suggest that fatty liver index is not elevated in young adult offspring of women with type 1 diabetes. Further research on whether pregestational type 1 diabetes in pregnancy affects offspring’s nonalcoholic fatty liver disease risk is warranted.

## Background

The accumulation of excess fat in the liver without a secondary cause of origin, such as alcohol consumption or viral infection, is the characteristic hallmark of nonalcoholic fatty liver disease (NAFLD) [1]. The global burden of NAFLD is excessive: it is estimated that over one-third of the adult population is affected by NAFLD [2]. Accumulating evidence suggests that the development of NAFLD could originate from exposure to maternal diabetes and obesity during fetal development [3, 4].

The fatty liver index (FLI) is a noninvasive risk calculator, which includes both liver enzymes and clinical characteristics for the screening of NAFLD [5]. It has previously been shown that offspring born to women with type 1 diabetes have higher FLI in late adolescence compared to offspring born to women without diabetes [6]. Whether a similar association is present in adulthood, however, is not known.

Therefore, the objective of this study was to compare FLI between offspring of women with type 1 diabetes and offspring of women without diabetes in young adulthood.

## Methods

### Study participants

A more detailed description of the study population has been published previously [7]. Briefly, between 1996 and 2000, we identified 238 singleton offspring of women with type 1 diabetes, born at the Department of Obstetrics and Gynecology, Helsinki University Hospital, Helsinki, Finland. From the Finnish Medical Birth Register, which gathers data on live births and stillbirths from all maternity hospitals, we identified 476 singleton age- and region-matched offspring of women without diabetes, born at maternity hospitals within the hospital district of Helsinki and Uusimaa, Finland. Since data on the matched offspring were obtained from a national register, they can be considered as representative of the background population.

### Clinical study

In 2019, we invited all identifiable study participants to a clinical follow-up study, consisting of laboratory tests, clinical measurements, and self-reported questionnaires.

After the exclusion of individuals with missing data (n = 10), 153 white Finnish offspring consisting of 70 offspring of women with type 1 diabetes and 83 offspring of women without diabetes agreed to participate.

### Laboratory assessment

After overnight fasting, blood was drawn for the following analyses: glucose, HbA_1c_, insulin, cholesterol, LDL, HDL, triglycerides, alanine transaminase, aspartate aminotransferase, and gamma-glutamyl transferase. A 2-hour 75-g OGTT was performed according to WHO’s guidelines to assess glucose tolerance. The HOMA-IR and fasting insulin were used to approximate insulin sensitivity in all study participants.

### Clinical assessment

Trained study nurses performed all the clinical measurements.

Height was measured to the nearest 0.1 cm in light indoor clothing without shoes, using a telescopic measuring rod (SECA Gmbh&co, Germany). Waist circumference (WC) was measured with a tape measure (m) placed midway between the iliac crest and the lowest rib. Waist-to-height ratio (WHtR) was calculated as WC (m) / height (m). Body fat (kg) and body weight (kg) were measured using the InBody 720 body composition device (InBody 3.0, Biospace, South Korea) without socks on, rounded to the nearest 0.1 kg. BMI was calculated as body weight (kg) / height (m^2^). Body fat (%) was calculated as body fat (kg) / weight (kg). BP was measured from each participant’s right arm with a standardized BP monitor (M6 AC, Omron Healthcare Co. Ltd., Japan), after 15 min of rest. The mean value of three BP measurements was used in the data analysis.

### Noninvasive assessment of NAFLD

The FLI was calculated according to Bedogni et al. [5]:

$$FLI = \left({e}^{0.953*loge\left(triglycerides\right) + 0.139*BMI + 0.817*loge\left(GGT\right) + 0.053*AC ? 15.745}\right) / (1\hspace{0.17em}+\hspace{0.17em}{e}^{0.953*loge\left(triglycerides\right) + 0.139*BMI + 0.718*loge\left(GGT\right) + 0.053*AC ? 15.745}) * 100$$.

It has previously been shown that a FLI < 30 can be used to rule out NAFLD, whereas a FLI ≥ 60 can be used to define NAFLD.

### Questionnaires

Self-administered questionnaires were used to gather information on general health, chronic diseases, and lifestyle behavior, including smoking. None of the study participants reported to have a medical history of liver diseases. The Alcohol Use Disorders Identification Test was used to identify unhealthy alcohol consumption. Physical activity was assessed according to the standardized Kuopio Ischaemic Heart Disease questionnaire [8]. Each type of exercise is assigned a Metabolic Equivalent of Task (MET)-value, where one MET (1 MET = 3.5 ml O_2_/kg/min) is roughly equivalent to the energy expenditure of metabolic state at rest (e.g., sitting quietly). Thus, physical activity was calculated as the sum of each activity’s MET-value, duration, and frequency, and reported as MET-hours (MET-h) per week.

### Statistical analyses

The data are presented as means with standard deviations (SD), medians with interquartile ranges (IQR; 25th and 75th percentiles), or frequencies with percentages. The two groups were compared with the t test or permutation test, for continuous variables, and Pearson’s chi-square test or Fisher’s exact test, for categorical variables. The distribution of FLI was compared between the groups by an Epps-Singleton two-sample empirical characteristic function test.

## Results

Table 1 provides the clinical characteristics of the study population. The mean age for offspring of women with type 1 diabetes was 20.4 (SD 1.6) years and 20.4 (SD 1.6) years for offspring of women without diabetes, respectively. Sex distribution was similar in both groups.

**Table 1:** Clinical characteristics of the study population (N = 153) divided into offspring of women without diabetes and offspring of women with type 1 diabetes

	Offspring of women without diabetes, N = 83	Offspring of women with type 1 diabetes, N = 70	P-value
Women, n (%)	55 (66)	45 (64)	0.80
Age, years, mean (SD)	20.6 (1.6)	20.4 (1.6)	0.45
Body mass index, kg/m^2^, mean (SD)	24.0 (4.8)	24.5 (4.6)	0.58
Body fat, %, mean (SD)			
Men	17.7 (7.1)	19.3 (10.8)	0.51
Women	30.1 (9.2)	33.0 (7.8)	0.091
Blood Pressure, mmHg, mean (SD)			
Systolic	119 (10)	117 (12)	0.46
Diastolic	74 (7)	74 (9)	0.84
Mean arterial pressure	89 (7)	88 (9)	0.65
Fatty Liver Index, score, median (IQR)	7.9 (3.5;21.5)	8.7 (4.3;36.5)	0.59
Waist-to-height ratio, mean (SD)	0.41 (0.08)	0.42 (0.08)	0.73
**Laboratory tests**			
Alanine transaminase, U/L, median (IQR)	21 (14;27)	19 (15;26)	0.77
Aspartate aminotransferase, U/L, median (IQR)	22 (20;27)	24 (21;28)	0.17
Gamma-glutamyl transferase, U/L, median (IQR)	13 (9;18)	14 (10;17)	0.88
Glucose, mmol/l, mean (SD)			
0 h	5.40 (0.43)	5.39 (0.41)	0.84
2 h	5.61 (1.43)	5.83 (1.67)	0.39
HbA_1c_, mmol/mol, mean (SD)	32.6 (2.2)	32.7 (2.6)	0.70
HbA_1c_, %, mean (SD)	5.13 (0.20)	5.15 (0.24)	0.70
HOMA-IR, mmol/L, mean (SD)	2.58 (1.80)	2.70 (2.24)	0.71
Insulin 0 h, mU/l, mean (SD)	10.6 (6.6)	11.0 (8.7)	0.72
Total cholesterol, mmol/L, mean (SD)	4.30 (0.65)	4.36 (0.72)	0.62
LDL, mmol/L, mean (SD)	2.54 (0.65)	2.57 (0.71)	0.80
HDL, mmol/L, mean (SD)	1.56 (0.37)	1.59 (0.38)	0.64
Triglycerides, mmol/L, mean (SD)	0.96 (0.44)	0.89 (0.53)	0.34
**Questionnaires**			
Alcohol Use Disorders Identification Test (AUDIT), points, mean (SD)	3.8 (2.3)	4.2 (2.2)	0.29
Metabolic Equivalent of Task per week, MET-h/week, mean (SD)	36.6 (44.6)	30.7 (28.8)	0.34
Smokers, n (%)	21 (25)	14 (20)	0.44

**Fig. 1:**
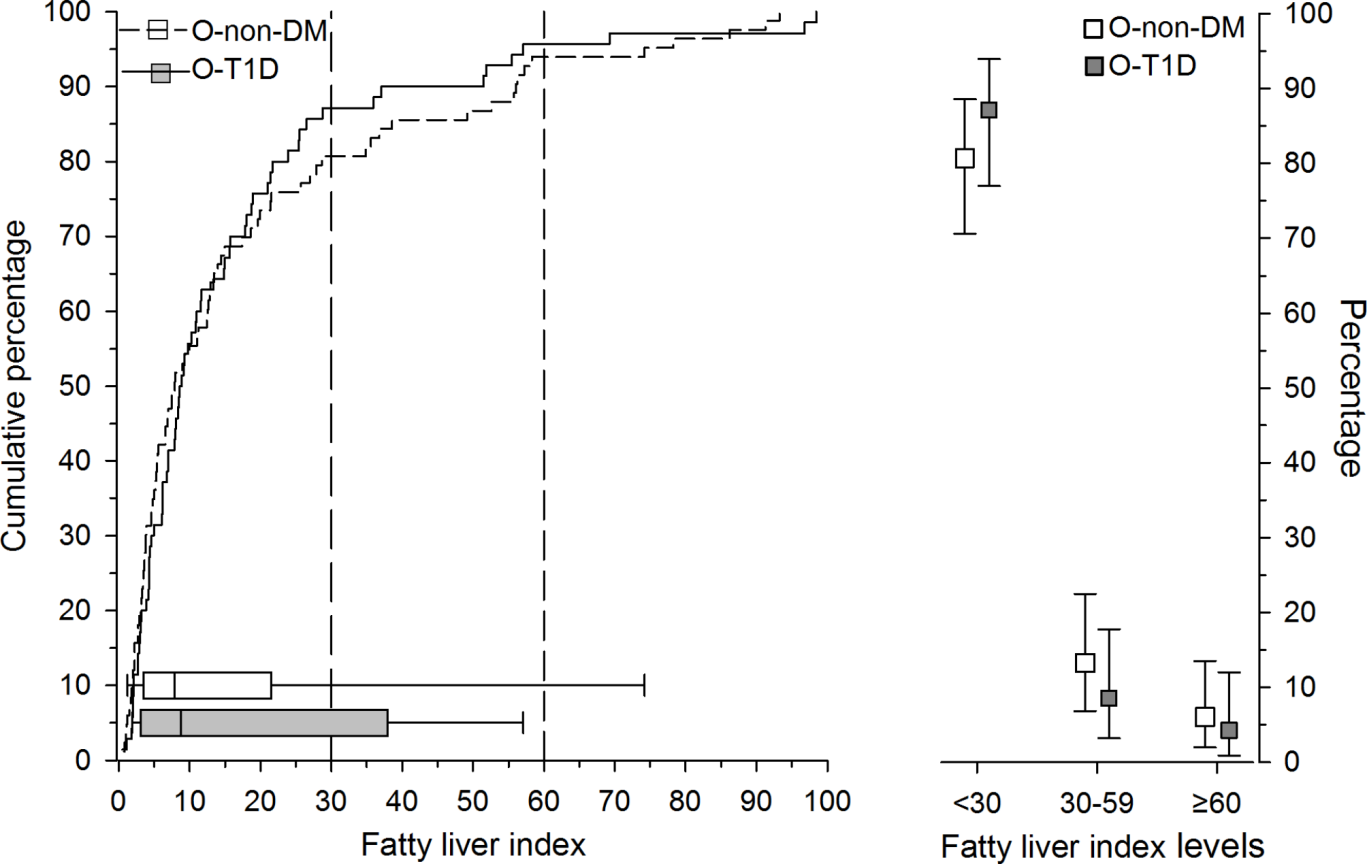
On the left, we present the cumulative percentage of FLI in offspring of women without diabetes (O-non-DM, white bars) and in offspring of women with type 1 diabetes (O-T1D, black bars). Box-and-whiskers plot shows median and interquartile range, and whiskers indicate 5th and 95th percentiles. On the right, we have divided the percentage of the study population according to fatty liver index levels. FLI ≥ 60 indicates nonalcoholic fatty liver disease

The differences between the groups regarding median FLI were statistically non-significant O-T1D 8.7 (IQR; 4.3–36.5) vs. O-non-DM 7.9 (IQR; 3.5–21.5) (p = 0.59). Moreover, as plotted in Fig. 1, the distribution of participants according to FLI was nearly identical for offspring of women with type 1 diabetes and offspring of women without diabetes. Lastly, dividing FLI according to relevant threshold values, i.e., rule-out (FLI < 30) and rule-in (FLI ≥ 60), showed no differences between the groups (Fig. 1).

We observed no differences between the groups regarding any metabolic characteristics: BMI 24.5 kg/m^2^ vs. 24.0 kg/m^2^, fasting glucose 5.39 mmol/L vs. 5.40 mmol/L, fasting insulin 11.0 mU/L vs. 10.6 mU/L, HOMA-IR 2.70 mmol/L vs. 2.58 mmol/L, systolic BP 117 mmHg vs. 119 mmHg, total cholesterol levels 4.36 mmol/L vs. 4.30 mmol/L. Also, we did not find any statistical difference regarding signs of abdominal obesity: WHtR was 0.42 for offspring of women with type 1 diabetes and 0.41 for offspring of women without diabetes. Furthermore, no differences in liver enzymes were detected between between the groups. Data received from questionnaires revealed no significant differences regarding lifestyle factors between offspring of women with type 1 diabetes and offspring of women without diabetes.

## Discussion

This study revealed no differences between young adult offspring of women with type 1 diabetes compared to offspring of women without diabetes for FLI.

Our findings are in contrast to those reported by Knorr et al. [6]. Our smaller sample size and slightly older study population could be among the factors explaining the differences. Furthermore, our results might be influenced by selection bias of participants with optimal lifestyle behavior; the characteristics of our study population differs regarding nearly all metabolic risk factors, as compared to Knorr et al. [6]. Also, albeit speculative, the precise diet incorporated in the lifestyle of maternal type 1 diabetes management might have impacted the familial eating behaviors on a broader scale, resulting in a healthier lifestyle in offspring.

Increasing evidence from observational studies have observed that the development of NAFLD could originate from *in utero* programming after exposure to maternal fuels [3, 4, 6]. Recently, however, a meta-analysis concluded that the association of maternal pregestational diabetes with offspring’s NAFLD is unclear, since many of the included studies did not adjust for important confounders, such as maternal BMI [3]. Although we were unable to adjust for confounding maternal characteristics, the women with type 1 diabetes in the present study have been published previously and 67% of the mothers were reported to have a pre-pregnancy BMI < 25 kg/m^2^ [9]. On the other hand, in 2016 Patel et al. found that maternal diabetes/glycosuria was associated with a 6.7-fold risk of ultrasound diagnosed NAFLD in adolescents (mean age 17.8 years) compared to mothers without diabetes or glycosuria [10]. Although the number of mothers with existing diabetes was low, this finding was adjusted for offspring’s fat mass and maternal BMI, which suggests a role for early exposure to maternal hyperglycemia [10].

In theory, the risk of lean NAFLD could still persist, however, the American Gastroenterological Association recommends that screening should not be performed in the general population [11]. Interestingly, both FLI and WHtR have been observed to be the best clinical characteristics for determining NAFLD in both lean and obese individuals in a Chinese population [12]. As graphically presented in Fig. 1, a value of FLI ≥ 60 (i.e., define NAFLD) did not distinguish between the groups. Thus, when combining the insignificant differences in FLI levels, metabolic characteristics, anthropometric measures, and lifestyle behavior between the study groups – it would suggest that the risk of NAFLD in young adult offspring of women type 1 diabetes is low.

The strength of this study includes detailed clinical assessment of the study participants. Our study involving age- and region-matched offspring in young adulthood adds to the perspective on the developmental origins of NAFLD. A clear limitation of this study is the small sample size and low participation rate. This might have led to selection bias, i.e., only individuals interested in their health metrics might have been more eager to attend this voluntary clinical study. The FLI is also a limitation and other imaging modalities would have been more appropriate for diagnosing NAFLD. Finally, maternal characteristics in pregnancy were not available for data analysis.

## Conclusion

In conclusion, we could not find any differences regarding FLI between offspring of women type 1 diabetes and offspring of women without diabetes in young adulthood. Well-designed large prospective studies are needed to elucidate the gaps regarding the relationship on fetal programming of NAFLD.

## Data Availability

The data used in this study are not available to the public due to legislative reasons.

## List of Abbreviations

FLI: Fatty liver index
NAFLD: nonalcoholic fatty liver disease
WHtR: Waist-to-height ratio
